# Collision tumour of the stomach with a cancer to cancer metastasis: a case report

**DOI:** 10.1186/1757-1626-1-63

**Published:** 2008-07-28

**Authors:** Alexandros Strofilas, Ioannis G Dalianoudis, Emmanuel E Lagoudianakis, Michael Genetzakis, Dimitrios Tsekouras, John Chrysikos, Nikolaos Koronakis, Vaggelogiannis Katergiannakis, Andreas Manouras

**Affiliations:** 1 First Department of Propaedeutic Surgery, Hippocrateion Hospital, Athens Medical School, Athens, Greece; 2 Second Department of Surgery, 417 NIMTS, Athens, Greece

## Abstract

**Introduction:**

Coexistence of a primary gastric lymphoma and a gastric adenocarcinoma is a rare event. The diagnosis is suspected after the pathologic examination of the endoscopic biopsies and definitely documented with the examination of the surgical specimen.

**Case presentation:**

We are presenting a rare case of a 77-year-old Greek man with epigastric pain of one and a half month duration, nausea, anorexia and weight loss. The pathologic examination of the endoscopic biopsies and a lymph node biopsy excised at laparotomy, presented the interpenetration of synchronous occurring primary gastric lymphoma and a gastric adenocarcinoma with a documented cancer to cancer metastasis.

**Conclusion:**

Prognosis of these rare tumours is largely dependent on the stage of the adenocarcinoma at presentation but due to lack of large series there are no data on the biological behavior of these tumours in comparison to adenocarcinoma.

## Introduction

Gastric adenocarcinoma is one of the most common gastrointestinal malignancies with primary lymphoma to account for 1–7% of all gastric neoplasms. The coexistence of a gastric adenocarcinoma and a primary gastric lymphoma is a rarely observed event with few reports in the literature [1-4]. Still only recently a possible causal relationship between those different malignancies has been suggested focusing on the role of the helicobacter pylori infection [3]. Besides helicobacter pylori infection Epstein Barr Virus has been reported in several cases of gastric adenocarcinoma and primary gastric lymphoma [5].

Collision tumors are thought to arise from interpenetration of two synchronous juxtaposed malignancies. Collision tumours are distinct entities from composite tumours which are characterized by two divergent lineages originating from the same neoplastic clonal proliferation.

We are presenting here a rare case of "a" collision tumour of the stomach with a lymphomatous perigastric node invaded by the gastric adenocarcinoma (cancer to cancer metastasis).

## Case presentation

A 77-year-old man from greece was admitted to our department with epigastric pain of one and a half month duration, nausea, anorexia and weight loss. The physical examination was negative. The laboratory work-up revealed a mild anemia and thrombocytopenia. The gastroscopy showed a 3 × 3 cm ulcerative lesion adjacent to the cardia infiltrating the surrounding tissues. The gastric mucosa appeared red, friable and with superficial erosions. The duodenum was normal. Multiple biopsies were taken from the stomach. The computed tomography(CT) scan examination demonstrated a thickening of the wall of the stomach and multiple enlarged periaortic lymph nodes.

**Figure 1:**
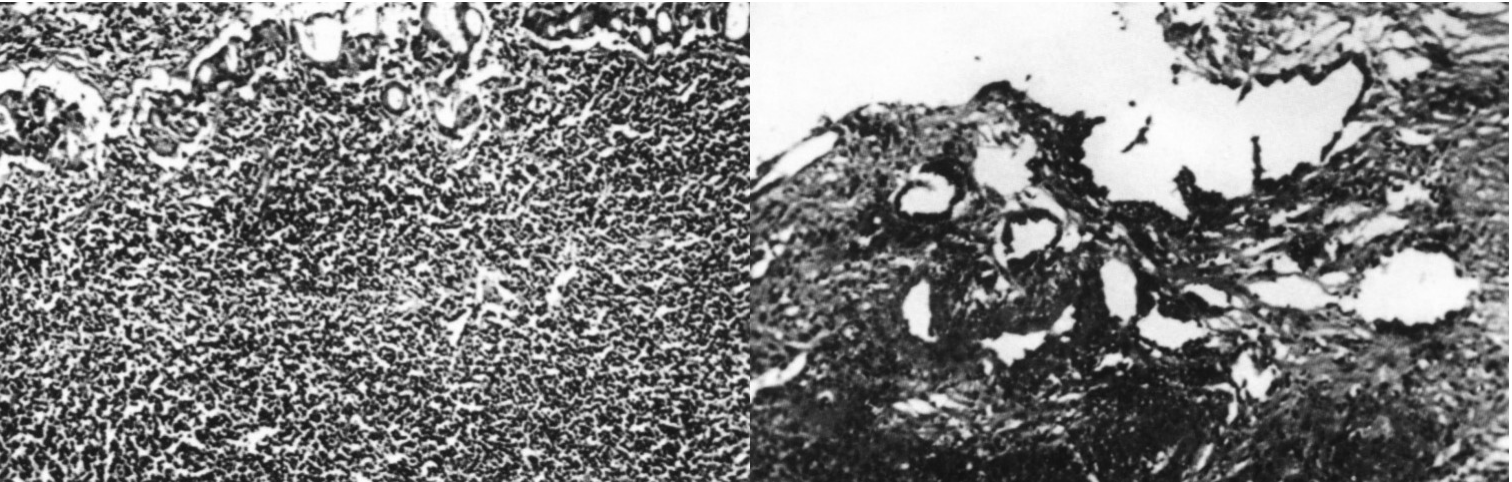
**Surgical specimen biopsy showed dense infiltration of the gastric mucosa from small and medium size lymphocytes (Left photo H-EX250).** Another site of the same specimen showed the presence of a tubular adenocarcinoma (Right photo H-EX125).

The pathologic examination of the endoscopic biopsies showed intestinal metaplasia and a dense lymphocytic infiltration of the mucosa giving the impression of a lymphoma. In a particular tissue fragment, anomalous adenomatous malformations with dysplastic epithelium were noted, suggesting the existence of a gastric adenocarcinoma (Figure 1).

At laparotomy, an invasive gastric tumour with multiple metastatic lymph nodes was found. Extent of the tumour prevented surgery with curative intend and a palliative gastrojejunostomy was created. A lymph node was excised for biopsy and the abdomen was closed.

**Figure 2:**
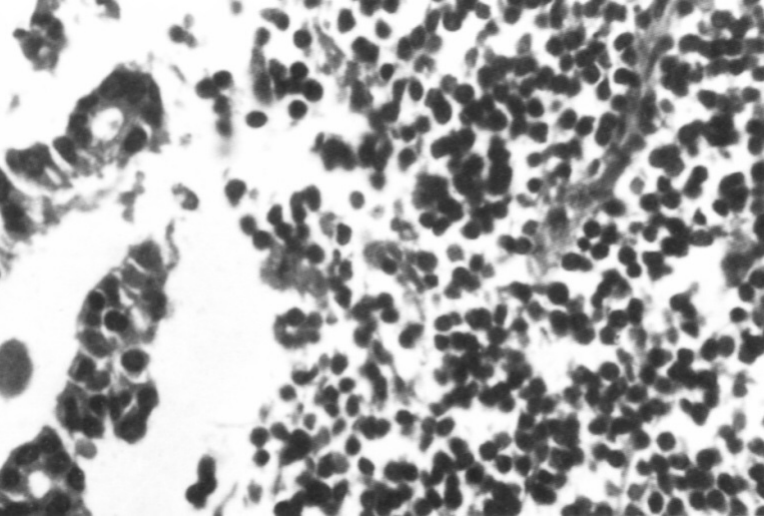
Lymphomatous lymph node with focal infiltration by an adenocarcinoma.

Subsequent pathologic examination of the removed lymph node showed total infiltration from a low grade lymphoma; with a limited metastatic infiltration from an adenocarcinoma noted in the same specimen (Figure 2).

The postoperative course of the patient was uneventful. He was discharged on the ninth postoperative day and referred for chemotherapy.

## Conclusion

While in our case histology showed the two primaries to interpenetrate each other still collision tumour is considered for two distinct primaries involving the same organ [6] even with an equivocal transition zone found between them [7]. Cancer to cancer metastasis referred to the metastatic site, commonly a node, where adenocarcinoma invades the lymphomatous tumour deposits [8].

Referring to the pathological features of gastric collision tumours reported in the literature a more frequent occurrence of the intestinal type adenocarcinoma in association with malt lymphomas is noted clearly setting the issue of the possible role of helicobacter pylori in the tumorigenesis of both synchronous malignancies. The latter hypothesis has been proposed on the basis of certain morphologic features: the carcinoma is frequently an early gastric cancer, well differentiated and less extensive than the lymphoma and the frequency of the coexistence is referred to be 0.36% in published series which is higher than expected by chance and strengthens the hypothesis of common etiopathogenesis [2,3]. The cases of "a" collision tumour frequently show an intestinal type early gastric tumour, relatively small with respect to the predominant lymphomatous proliferation [4]. The association of gastric dysplasia and mucosa associated lymphoid tissue(MALT) lymphoma has also been reported [3]. The relationship of helicobacter pylori with MALT lymphoma has been hypothesized by the observation that antibiotic therapy against this bacterium has been shown to cause lymphoma regression, at least in cases at an early stage [9]. Helicobacter-pylori have been found in 78% of synchronous double tumours, implicating a possible etiologic role in concurrent tumours [3].

On the other hand, the occurrence of a gastric adenocarcinoma after treatment for primary gastric lymphoma is more frequent. The metachronous adenocarcinoma can be considered as a late complication of the cancer therapy; indeed, the occurrence of an adenocarcinoma in a gastric stump or after irradiation has been extensively described [10].

The prognosis of patients with "a" collision primary gastric lymphoma and adenocarcinoma has not been clarified due to the lack of large series and long-term follow-up observations. However, it seems that the survival probability is similar to that for patients with gastric adenocarcinoma without lymphoma and significantly worse than that for patients with MALT-type gastric lymphoma without adenocarcinoma [10].

## Abbreviations

CT: Computed Tomography; MALT: Mucosa Associated Lymphoid Tissue.

## Competing interests

The authors declare that they have no competing interests.

## Authors' contributions

AS contributed to manuscript conception, research, acquisition of data, drafting and writing of the manuscript. IGD contributed to manuscript conception, research, acquisition of data, drafting and writing of the manuscript. EEL contributed to manuscript conception, research, acquisition of data, drafting and writing of the manuscript. MG contributed to manuscript conception, research, acquisition of data, drafting and writing of the manuscript. DT contributed to manuscript conception, research, acquisition of data, drafting and writing of the manuscript. JC contributed to organizing, drafting and critical review of the manuscript. NK assisted in the operation and contributed to writing of the manuscript. VK contributed to organizing and drafting of the manuscript, and critically revised the manuscript. AM assisted in the operation and contributed to critical review of the manuscript. All authors read and approved the final manuscript.

## Consent

Written informed consent was obtained from the patient for publication of this case report and any accompanying images. A copy of the written consent is available for review by the Editor-in-Chief of this journal.
